# Supplementary material for: Are carbon emissions trading and green financial instruments synergistic? -Comprehensive quantitative research based on content analysis
Source: PLoS One. 2024 Mar 7;19(3):e0298601. doi: 10.1371/journal.pone.0298601 (PMC10919601; doi:10.1371/journal.pone.0298601)
Supplement: S1 Data — (ZIP) [file pone.0298601.s001.zip › Data/地级市Z_F工作报告.zip!/┴╔─■/╙¬┐┌╩╨/└┤╫╘╣1⁄2╓┌║┼í╛┬φ┐╦╩2╛▌═°í┐_2005.docx]

各位代表：

　　我受市人民政府委托，向大会作政府工作报告，请予审议，并请政协委员和列席会议的同志提出意见。

　　一、２００４年工作的回顾

　　２００４年是实施老工业基地振兴战略的开局之年。一年来，在市委的正确领导下，我们以“三个代表”重要思想为指导，认真贯彻党的十六大和十六届三中、四中全会精神，全面落实科学发展观，经过全市人民的共同努力，圆满完成了市十三届人大二次会议确定的工作任务。全年实现地区生产总值３１８．３亿元，比上年增长２１．２％；固定资产投资１５８亿元，增长５２．５％；财政一般预算收入（新口径）１２．６亿元，增长２６．８％；城市居民人均可支配收入８１２８元，增长１１．１％；农民人均纯收入３８６８元，增长８．６％。

　　强化农业基础地位，农村经济全面发展。认真落实国家惠农政策，协调发展农村各业。全年完成第一产业增加值３６．８亿元，增长９％。粮食总产量达到６０．５万吨，增长２８．６％，订单农业面积７５万亩。“两水一牧一菜”等特色产业取得长足发展，水产品产量３４．６万吨，增长６．８％，工厂化养殖面积１８万平方米，增长１倍；水果总产量４６．６万吨，增长２０．４％；肉蛋奶总产量２１．５万吨，增长１９．９％；蔬菜总产量５７．６万吨，增长２．１％。４１户规模以上龙头企业实现销售收入１９．３亿元，增长１４．１％。１０个现代农业园区累计完成投资７．７亿元，吸纳劳动力１．５万人。治理水土流失面积１１．６万亩，新修和加固河道堤防１７６公里。完成人工造林９万亩，植树２６０１万株。实施人工增雨１２次，增加生态降水１亿多立方米。

　　加速产业和产品结构调整，工业经济运行质量不断提高。继续坚持“工业强市”思路，通过改革、改组、改造，促进了工业经济的快速增长。全年完成第二产业增加值１７０．１亿元，增长３０．６％。规模以上工业总产值４１１．４亿元，增长４０．７％；实现利税３０．５亿元，增长１９．９％。充分依托现有工业基础，大力发展冶金、石化、建材等产业，加速了我市工业由以轻纺为主向重化工与轻纺并重转变，轻重工业比例调整到３１．７∶６８．３。实施企业改革和资产盘活项目６５个，分流富余人员１．７万人，盘活存量资产４．９亿元。实现工业固定资产投资８３．９亿元，增长６６．１％；净增规模企业２００户。全年开发新产品６７１种，实现新产品产值４０．２亿元。１０个工业园区投资总额达到３７．３亿元，增长１．３倍；规模以上工业产值完成１２５．５亿元；实现利税１３．４亿元。民营经济快速发展，实现增加值１９８亿元，占全市经济总量的６２．２％；规模以上工业民营企业达到８１３户，占总数的９０％，产值超亿元的民营企业达到４２户。

　　积极构建现代服务业体系，第三产业取得长足发展。稳步提升传统服务业，加快发展新兴第三产业，规范市场经济秩序，进一步搞活商贸流通。全年完成第三产业增加值１１１．４亿元，增长１２．７％。社会消费品零售总额达到９３．４亿元，增长１４．５％。邮电通信业务总量１５．８亿元，增长１３．４％；保险费总收入７．５亿元，增长８．７％；旅游总收入１３．２亿元，增长１５．８％。

　　深入开展“招商引资年”活动，开放型经济实现新突破。坚持“引进来”、“走出去”相结合，不断加大外引内联力度。全年批准外商投资企业１４６家，合同外资额７．５亿美元，增长５８％；外商直接投资３．３亿美元，增长３４％；引进内资７１．３亿元。开工建设投资５００万元以上项目５４５项。大项目开发取得历史性突破，鞍钢的５００万吨钢、华能电厂二期工程、五矿中板二期改造工程、嘉晨集团焦化和伊斯帕特镀锌板等一批重点项目顺利实施。对外贸易不断扩大，进出口总额达到１１．８亿美元，增长３３％，其中出口７．７亿美元，增长２６．９％；境外劳务输出２９００人，增长２８％。先导区带动作用突出，开发区实现外商直接投资９５４６万美元，增长４６．５％；出口创汇３．５亿美元，增长２４．４％。高新区完成基础设施投资６．２亿元，进区企业１５６户，实现技工贸总收入１１．２亿元，增长３．１倍。港口建设步伐加快，２０万吨级矿石码头、成品油及液体化工码头等工程顺利完工。实现港口吞吐量５９７７万吨，增长４９．１％；集装箱运量５８．３万标箱，增长４５％。

　　加强重点基础设施建设，城乡面貌明显改观。积极推进城市经营，依法规范土地和矿业市场秩序，全年出让土地使用权４１宗、１３８．８万平方米，获取出让金４．１７亿元；出让矿山探矿、采矿权７０宗，获取出让价款５７７９万元。城市开发改造保持旺盛势头，全年商品房建筑面积１９３万平方米，改造棚户区５０万平方米，房产交易额１０３亿元，我市荣获全国房地产交易规范化管理先进单位。社区建设力度加大，投资２２８２万元，新建、扩建社区办公楼３５处。建设农村公路网６００公里，超额完成年度计划。年初确定为市民办的２０件实事已完成１６件，滨海大道建设等４项跨年度工程正按计划推进。改善小街小巷环境，集中整治市容市貌，城市美化、绿化、亮化、净化水平进一步提高，“绿叶杯”竞赛进入全省先进行列。城市环境质量不断改善，大气质量基本稳定在二级标准。

　　以增收节支为主线，财政金融状况有所改善。坚持依法理财，依法治税，促进了财政收入的大幅度增长。全口径财政收入完成３５．１亿元，增长２１．３％。全口径税收３２．８亿元，增长２１．３％。一般预算支出３０．２亿元，增长２２．３％。金融机构各项存款余额３３８亿元，增长１７．８％；各项贷款余额３０８亿元，增长１２．３％；不良贷款率比年初降低４．８个百分点。争取国债资金及配套贷款４．５亿元，使４６个项目得以顺利实施。我市被国家开发银行确定为全省唯一的中小企业贷款试点城市，共引进开行及外埠银行贷款１０．５亿元。积极化解金融风险，顺利完成了财政证券公司兑付和辽宁证券营口分公司托管经营，收购个人债权近１０亿元；深化农村信用社改革，提前完成了增资扩股任务。

　　认真做好就业和社会保障工作，人民生活水平稳步提高。积极构筑城乡统筹的就业格局，全年新增就业７．９万人，登记失业率控制在５．５％。培训下岗失业人员３．６万人，就业率达到６０％以上。安置大龄特困人员就业５９００人。企业养老保险参保人数比去年净增５．６万人，参保人员总数达到３０．７万人，养老保险缴费比例由２５．５％降至２０％；医疗保险新增参保人员３．２万人，参保人数达到２７．５万人。充分发挥最低生活保障作用，累计为７万名城镇困难居民发放低保金７７５４万元，为１．４万名农村困难居民发放低保金５１７万元。政府出资１．７亿元，安置动迁户４１２３户；政府承债５８７８万元，解决了２．４万名破产、改制和特困企业退休职工进医保问题。深入开展扶贫帮困活动，筹集扶贫帮困款物２０００万元，救助城乡特困群众５．８万户次。投入资金５００万元，完成农村特困户建房１７５户。

　　加快“科教兴市”步伐，各项社会事业全面进步。积极推进科技进步与创新，实施高新技术产业化项目１０３项，高新技术产业产值达到５３．１亿元，增长２８．９％。镁质非金属材料项目被列入“十五”国家重大科技攻关计划，镁质材料规模以上企业实现工业产值９０．３亿元，占全市规模以上工业的２１．９％。引进和推广农业新技术１１项、新品种４１个。大力发展教育事业，进一步推进农村义务教育，加快了“双高普九”进程。中、高职专招生分别增长１８．２％和４．５％，就业、升学率达９０％以上。改造农村中小学校４８所，消灭危房３．２万平方米，７０％的农村初中进入省标准化学校行列。投资２．５亿元完成了高中学校布局调整，又有２所高中进入省示范性高中行列。广泛开展专业艺术和群众文化活动，丰富和活跃了城乡群众的文化生活。文化市场得到进一步规范，取缔音像制品无证经营场所５３家，停业整顿网吧５９家。广电事业不断发展，政府投资解决了盖州东部山区３０多万群众看不到营口电视节目问题。公共卫生体系建设步伐加快，疾病预防控制、妇幼保健、卫生执法监督等项工作扎实开展。人口和计划生育工作出现新局面，人口自然增长率控制在１‰以内。群众体育活动广泛开展，竞技体育水平进一步提高。

　　切实加强民主法制建设，提高了政府决策的民主化、科学化水平。各级政府自觉接受人大及其常委会的依法监督、政协的民主监督和人民群众的社会监督，认真执行人大及其常委会的决议、决定，全年共办理人大代表建议２２６件、政协提案２１９件，办复率１００％，满意率达到９５％以上。建立了市长与人大代表“直通车”制度，共收到代表建议３１件，做到１００％批复，吸收采纳２４件。加强与各民主党派、工商联、人民团体及各界人士的联系，党的民族宗教和侨务政策得到进一步落实。深入开展“四五”普法活动，“法治营口”建设全面启动。以贯彻实施《行政许可法》为契机，积极推进依法行政，全年共废止规范性文件１７６１件，保留市本级行政许可项目２４１项，行政执法大检查和“双清理、一规范”活动取得明显成效。为了改善投资环境，成立了行政效能投诉中心，受理各种行政不作为和乱作为投诉案件８９件，已办结８６件。扎实推进学习型城市建设，广泛开展群众性精神文明创建活动，巩固了省级文明先进市的创建成果。切实加强公安基础工作和社会治安综合治理，全年破获各类刑事案件４０７６起，保持了良好的治安环境。继续开展与“法轮功”等邪教组织的斗争。切实做好安全生产和信访工作，有力地维护了社会稳定。大力加强廉政勤政建设，机关作风有所改善。人防工作取得新进展，国防教育和民兵、预备役工作扎实推进，双拥成果得到巩固。

　　各位代表，过去的一年，是我市经济结构调整力度最大、开发项目最多、增长幅度最高、实现效益最好的一年，地区生产总值、全社会固定资产投资、全口径财政收入等多项指标已提前完成“十五”计划。在这一年里，面对复杂的形势和诸多的困难，全市上下齐心协力、开拓进取，经济和社会发展取得了丰硕成果。这是市委正确领导的结果，是市人大依法监督和政协民主监督的结果，是社会各界和驻军、武警官兵及中省直单位大力支持的结果，也是全市广大干部群众团结奋斗的结果。在此，我代表市人民政府表示衷心的感谢！

　　在肯定成绩的同时，我们也清醒地认识到，全市经济和社会发展中仍然存在一些不容忽视的问题：农业投入不足，农村工业化、产业化和城镇化程度低，社会化服务体系不健全。工业经济总量小，支撑力强的大项目、大企业少，高新技术产业发展缓慢。对外开放的水平和质量不高，利用外资和外贸出口规模较小。财政收支矛盾突出，财力紧张状况没有得到有效缓解。就业和再就业压力仍然很大，部分低收入群体生活困难。政府职能转变相对滞后，一些部门仍存在着服务意识不强、工作作风不实、行政效率不高等问题。对此，在今后的工作中要采取有效措施，认真加以解决。

　　二、２００５年工作的主要任务

　　２００５年是实施老工业基地振兴战略的关键一年。按照市委九届九次全会的总体要求，政府工作的指导思想是：以邓小平理论和“三个代表”重要思想为指导，认真贯彻党的十六大和十六届三中、四中全会精神，紧紧抓住东北振兴和沿海开放的双重机遇，全面落实科学发展观，坚持以改革开放和科技进步为动力，以产业结构调整为主线，以环境建设为重点，突出抓好工业经济和县域经济，大力发展现代服务业，加速构建“一个平台、三大基地、五大产业”；坚持以人为本，进一步强化社会保障和就业工作，繁荣社会主义先进文化，不断提高城乡人民生活水平，促进国民经济和社会事业平稳、快速、健康发展，努力构建和谐营口。

　　２００５年主要奋斗目标是：地区生产总值３７８亿元，比上年增长１８％；固定资产投资２１０亿元，增长３３％；外商直接投资２．４亿美元（新口径），增长３７％；外贸出口１０亿美元，增长３０％；地方财政一般预算收入１４．９亿元，增长２０．３％；城市人均可支配收入８９４１元，增长１０％；农民人均纯收入４１８０元，增长８．１％；城镇登记失业率控制在５．５％以内。

　　为实现上述目标，要重点抓好以下十二个方面工作：

　　（一）深入开展“科学发展年”活动，用科学发展观统领政府工作全局

　　按照市委提出“科学发展年”的要求，把科学发展观贯穿于政府工作的各个领域、各个方面。

　　贯彻“五个统筹”的方针，促进经济社会全面、协调、可持续发展。坚持统筹城乡发展，实行以城带乡、以工促农，逐步改变城乡二元经济结构，加速城乡一体化进程。努力改善农业生产和农民生活条件，完善农村社会保障功能，缩小城乡差别。坚持统筹区域发展，通过增加投入、政策倾斜等措施，突出县级工业园区和中心城镇建设，支持经济落后市（县）、区和乡镇发展，促进共同繁荣。坚持统筹经济社会发展，在保持经济平稳快速增长的同时，把更多的注意力放到促进经济社会协调发展上来，加快科技、教育、文化、卫生、体育等社会事业发展，实现物质文明、政治文明、精神文明建设同步推进。坚持统筹人与自然和谐发展，正确处理经济建设、人口增长与资源利用、环境保护的关系，决不以浪费资源和破坏环境为代价，换取暂时的经济增长速度。加强生态环境建设，合理开发利用资源，大力发展循环经济，建设节约型社会。坚持统筹域内发展和对外开放，充分利用“两种资金”、“两种资源”和“两个市场”，在更加开放的环境中推进营口老工业基地全面振兴。

　　坚持以人为本，积极构建和谐社会。加强法律和制度建设，保障公民的平等权利和义务。重视人力资源的开发和利用，充分发挥人的积极性和创造性。把经济和社会发展与提高人民生活水平统一起来，不断满足人民群众物质和文化等方面的需求。努力提高人民群众的思想道德素质、科学文化素质和健康素质，促进人的全面发展。

　　按照统筹兼顾、突出重点的要求，妥善处理经济发展中的各种关系。正确处理当前与长远的关系，立足当前，着眼长远，全力推进结构优化和项目建设，协调发展生产要素，增强城市综合竞争力。正确处理三次产业的关系，在继续强化农业基础地位，不断提升服务业整体水平的同时，壮大工业经济实力，走“工业强市”之路。正确处理速度与效益的关系，加大固定资产投资力度，进一步做大经济总量，着力提高运行质量和效益，实现速度与效益同步增长。正确处理改革、发展和稳定的关系，坚持以改革促改造、促调整、促发展、保稳定，以新思路、新体制、新机制、新方式走出振兴营口老工业基地的新路子。

　　（二）加快结构调整和体制创新，积极推进工业化进程

　　坚持产业兴市、工业先行、项目为纲、统筹发展的方针，做大做强工业经济。

　　引导产业集群发展，构建特色产业区域。以打造“一个平台”、建设“三大基地”、发展“五大产业”为主攻方向，精心培育市场主体，重点发展现代物流、镁质材料、石油化工、新型建材、装备制造、黑色及有色金属、纺织服装等产业集群。按照“政府引导、市场运作、滚动开发、分步实施”的思路，加快推进工业园区建设，着力增强产业聚集、要素集约、环境承载和自我发展能力。全年工业园区固定资产投资、利用外资、规模以上工业产值、利税增幅均要高于全市平均值１５个百分点以上。

　　突出重大项目建设，打造骨干企业。加大跑项目、上项目工作力度，以５００万吨钢、华能电厂二期工程、２００万吨宽厚板、焦化、镀锌板等项目为重点，集中力量抓好１００个投资５０００万元以上的重大项目建设，努力营造良好的发展环境，确保重点项目早日竣工，早日投产，早日见效。

　　用高新技术改造传统产业，提高企业的技术创新能力。积极支持重点行业和企业建立技术创新体系，鼓励开发有自主知识产权的核心技术，推进高新技术产业化。全年实施科技研发项目６０项，推广新技术１００项。大力推进企业技术进步，从财政贴息、国债资金、土地利用等方面，强化对重点行业和重点企业技术改造的支持，全年完成投资１５亿元，新增产值４３．５亿元；开发新产品７５０种，实现新产品产值５０亿元。制定扶持名牌产品发展的政策，引导企业开发和培育名牌，进一步壮大人民大会堂牌卷烟、诺的斯卡牌钢琴等十大名牌产品，努力增加驰名商标数量，扩大知名产品生产规模。

　　继续深化企业改革，加速实现体制创新和机制创新。充分发挥市场对生产要素的配置作用，坚持以存量吸引增量、以增量激活存量，盘活国有企业资产。推进和规范企业公司化改造，完善法人治理结构，做好企业上市前的准备工作。深化国有企业内部分配、用工和人事制度改革，积极探索国有资产监管和经营的有效形式。规范国有企业产权交易，建立和完善劣势企业退出的市场机制，稳妥推进企业破产和重组，全年实施企业改革和资产盘活项目３０项，分流安置职工６０００人。强化国有资本管理，努力盘活非生产经营性国有资产。加强对民营企业的扶持和引导，鼓励民营企业以多种形式参与国有企业改革和重组，促进民营企业向规模化、集团化方向发展。

　　（三）推进农业和农村经济结构调整，切实解决“三农”问题

　　贯彻“多予、少取、放活”的方针，千方百计实现农民增收、农业增效，促进农村经济全面发展。

　　以市场为导向，积极发展高效农业。坚持用工业化理念谋划农业，实行区域化种植、规模化生产、产业化经营，不断优化农业和农村经济结构。落实最严格的耕地保护制度，提高粮食综合生产能力，粮食播种面积稳定在１３０万亩。按照“两水一牧一菜”的思路，优化农业区域布局，重点发展畜牧业和水产养殖业，逐步建立畜牧、水产、水果、蔬菜、林蚕五大农产品生产基地。坚持标准化生产，集中搞好资源、品牌和市场整合，大力发展高效农业、精品农业和创汇农业。建立健全农产品质量安全体系，积极推行农产品市场准入制度，无公害农产品生产面积达到５５万亩。

　　全力扶持龙头企业发展，提高农业产业化水平。大力发展农产品加工业，促进农产品由初级加工向精深加工转变，由资源消耗型向高效利用型转变。坚持种养加相结合、产供销一条龙、贸工农一体化的原则，积极引导和扶持龙头企业培育名牌产品、扩大规模，增强辐射带动能力。全年投资２亿元，新上、续建、改扩建旺发生猪饲养等投资５００万元以上项目２０个。积极发展农民专业合作组织，探索建立龙头企业与农户的新型利益联结机制，进一步完善粮食、蔬菜、水果等１０条产业链。全年完成投资２．５亿元，重点抓好西海等１０个农业园区建设。发挥龙头企业的集聚效应，促进小城镇建设，加快农村工业化和城镇化进程。

　　加强农业基础设施建设，改善农业生产条件。提高财政、信贷支农资金比重，积极吸引社会投资，多渠道增加农业投入。以治水治山为重点，搞好蓄水工程建设，全年兴修骨干水利工程３０项，治理水土流失面积９．２万亩。加快发展生态经济型林业，积极保护山林植被，大力实施荒山造林等工程，完成造林６万亩，植树１６００万株。

　　继续深化农村改革，落实党的惠农政策。认真贯彻《农村土地承包法》，全面做好农村土地二轮延包工作。坚持“依法、自愿、有偿”的原则，稳妥推进土地使用权流转，促进土地向适度规模经营方向发展。继续推进农村税费改革，着力化解农村债务，落实各项惠农政策，全部免征农业税，切实减轻农民负担。加强农业科技推广体系建设，完善四级服务网络。发展行业专业协会等服务型组织，壮大农村经纪人队伍，增强社会化服务功能。

　　（四）加大外引内联力度，大力发展开放型经济

　　充分发挥沿海开放优势，进一步提高利用外资的质量和水平，努力扩大外贸出口。

　　加快先导区建设，发挥“龙头”和“窗口”的辐射带动作用。开发区要充分发挥港口优势，千方百计引进大项目、大企业，增强对全市经济发展的带动能力，真正成为对外开放的先导区、新型工业化项目的集聚区和港口城市经济发展的示范区，全年地区生产总值、财政收入和实际利用外资等主要经济指标增长率确保３０％以上。高新区要利用土地储备丰富和靠近主城区的优势，完善技术创新机制，力争在基础设施建设、新技术应用、重大项目引进、利用外资、出口创汇等方面有新的突破，各项主要经济指标增长５０％以上。

　　坚持外引内联并重，扩大引进域外资金规模。按照内外并举、以外为主的方针，优化外资结构，拓宽招商领域。在积极有效地利用外资的同时，加强同国内经济发达地区的合作，吸引南资北移。以产权招商为重点，认真组织好国内外重大招商活动，创新招商方式，提高招商引资效率。强化招商基础工作，建立重大项目储备中心，搞好项目的筛选和包装，全年推出重点项目５００项。建立和完善招商引资项目的管理、考核和激励机制，强化跟踪服务，形成上下联动、全民招商的局面。

　　大力开拓国际市场，促进对外贸易持续增长。加强对出口企业的政策扶持，确保国家出口退税政策落实到位。充分利用老工业基地外贸资金、出口奖励基金和中小企业国际市场开拓资金，鼓励企业调整产品结构，扩大科技含量和附加值高、竞争力强的出口产品规模。引导企业积极参加国际商品交易会、博览会，大力开拓出口市场。认真组织好相关企业的反倾销应对，有效化解国际贸易摩擦。继续实施“走出去”战略，支持企业广泛开展跨国投资、国际工程承包，发展对外合作和劳务输出。

　　（五）加快亿吨大港建设，积极发展临港产业和沿海经济

　　继续实施“以港兴市”战略，举全市之力支持港口发展，加速打造港口经济平台。

　　进一步加快港口基础设施建设，全面提升港口功能。加大港口基础设施的投入，全年确保完成投资３０亿元，集中力量抓好鲅鱼圈港区四期和深水航道、疏港公路、疏港铁路等重点工程建设，争取开辟仙人岛港区，全面提升港口保税、物流和期货交割功能。不断拓宽投融资渠道，千方百计吸引外资、民资及信贷资金加快港口发展。积极参与东北亚航运中心建设，支持发展水运事业，争取开拓新的集装箱外贸航线。抓好营口港至韩国仁川的客货班轮运营，进一步提高经济效益。继续实施“大通关”工程，不断改善港口的管理与服务，提高集疏运能力。全年完成港口吞吐量７５００万吨，集装箱运量８０万标箱。

　　充分依托港口优势，大力发展临港产业。按照统一规划、分步实施的原则，研究制订土地使用、经营管理等相关政策，充分发挥港口的辐射和带动作用，加快发展以石化、冶金、建材、装备制造、修造船及船舶配套、矿产品加工、农产品加工为重点的临港工业。

　　加大对港口的支持力度，实现港口与城市的协调发展。继续加大对港口发展的政策支持，在生产要素配置上向港口倾斜，积极探索国有资本向港口建设积聚的有效途径。加快实施滨海大道工程，大力开发沿海经济带。以港口为龙头，统筹规划和建设，促进新老城区共同发展。

　　（六）提升传统第三产业，加速发展现代服务业

　　按照优化结构、提升水平的原则，进一步搞活商贸流通，大力发展新兴第三产业。

　　建立和完善市场体系，推进现代物流业发展。加快中国北方五金商贸城、营口华夏石材陶瓷城扩建等工程建设，培育一批联合经营的龙头企业。统筹规划和建设农产品区域性交易市场，发展产销直挂、配送经营、网上交易等营销形式。积极发展以原材料及商品批发配送、运输和仓储服务为主的物流企业，加快物流基地建设。

　　繁荣发展服务业，努力搞活商品流通。编制商业发展总体规划，积极引进国内外知名企业，促进服务业发展。调整优化服务业结构，大力发展连锁经营和社区服务业，实现传统商业形式与新兴业态的有机结合。

　　全方位开拓旅游市场，加快旅游业发展。编制旅游业总体规划，充分挖掘整合历史文化和自然资源，开发旅游产品，扩大对外宣传，调动社会力量搞好旅游资源开发。继续办好望儿山母亲节等活动，抓好开发区月亮湖公园等重要景点建设，不断拓展旅游服务领域。全年实现旅游总收入１４．５亿元，增长１０．２％。

　　（七）强化城乡规划、建设和管理，加快城市现代化进程

　　坚持市场化运作，加强城乡基础设施建设，积极构建现代城镇体系，逐步提高城市化水平。

　　实施高起点规划和高效能管理，进一步完善城市功能。突出规划的科学性和前瞻性，不断完善城市主要区片、城乡结合部和重点乡镇的规划编制，扩大控制性详细规划覆盖面。开发区要突出海滨城市特色，高起点做好城市扩张规划。对新上各类项目都要做好土地评审、环境评价、安全评价等前期工作。强化规划审批，加大监督力度，维护规划的严肃性和权威性。加强房地产开发后续管理和住宅区物业管理，完善住房公积金制度，进一步规范房地产市场。着力解决违章建筑、车辆占道、侵占绿地、垃圾治理等问题，建立城市公共管理的长效机制。

　　加强城乡基础设施建设，努力为人民群众办实事。按照量力而行的原则，全年集中力量办好１５件实事：（１）新建和改扩建农村公路４条、６６公里；（２）改造６０万平方米棚户区；（３）新建青花大街，扩建金牛山大街；（４）新建８处健身文化广场；（５）兴建１０处社区办公用房，改造７个城市住宅小区；（６）改造和维修市区小街小巷道路６万平方米；（７）新建中水回用设施；（８）完善城市环卫设施系统，新建和维修公厕１１０座；（９）建设农产品质量安全检验检测体系；（１０）改造１９所农村乡镇卫生院；（１１）改造２０所农村中小学校；（１２）易地建设市第二高中，新建市辽南育才学校；（１３）扩建西炮台遗址公园；（１４）新建市档案馆、图书馆和科技馆；（１５）新建市国防教育基地。同时，要抓好市中心医院、城市生活垃圾处理厂等跨年度实事工程建设，加快城市供水管网改造。

　　以争创全国环保模范城为目标，加强城市生态环境建设。大力实施城市绿化工程，全年市区植树１０万株，新增公共绿地２０公顷，人均公共绿地面积达到６．８平方米，城市绿化覆盖率达到３３．８％。加强生态保护与环境整治，搞好集中供热，有效治理水源、粉尘和噪声污染，全面改善城市环境质量。

　　以城市资产经营为重点，多渠道筹措城乡建设资金。按照政府调控、市场运作、依法管理的原则，进一步规范土地一级市场，完善土地招标、拍卖、挂牌和收购储备制度，提高土地利用率。加强矿产资源的管理和利用，继续推行探矿权和采矿权的有偿、有序出让。积极探索供水、供暖、污水处理、垃圾处理等城市资产的出让与合作。大力推进城市建设投资体制改革和公用事业改革，放开经营性和准经营性城建项目，鼓励社会资本参与城市建设和经营。争取和用好国家开行等金融机构贷款，力争有更多项目列入国债和各类基金项目计划。加快棚户区改造步伐，努力改善城乡环境和人居条件。

　　（八）改善财政管理与金融服务，确保财政金融平稳运行

　　坚持开源与节流并重的方针，继续深化财税、金融体制改革，增强对经济和社会发展的支撑能力。

　　进一步抓好财源建设，整合政府财力资源。采取财政贴息、政府资产抵押、国债转贷等多种政策手段，促进支柱产业、骨干企业、重大项目的快速发展，培育和壮大新兴财源。坚持依法治税，提高税收征管质量和水平，引导诚信纳税，打击偷、逃、抗、骗税行为。整合各类专项资金、非税收入、政府经营性资产、非经营性资产和政府资源性资产，合理配置财政资源，增加政府可控财力。

　　优化财政支出结构，提高理财用财水平。深化部门预算和“收支两条线”改革，全面推行财政国库集中收付制度，保证财政资金安全、规范、高效运行。推进政府债务预算管理，建立偿债基金，增强化解财政风险能力。严肃财经纪律，规范政府采购行为，最大限度地节约财政资金。按照构建公共财政框架的要求，严格执行收支预算，合理界定财政供养范围，集中财力保证重点支出。牢固树立过紧日子的思想，从严控制会议费、招待费、差旅费等一般性经费支出，提高财政资金使用效益。

　　构建社会信用体系，确保金融事业健康发展。强化社会信用意识，建设公共信用平台，逐步形成以政府信用为主导、企业信用为重点、个人信用为基础的信用体系。加强对审计、评估等中介机构的整顿，严厉打击恶意逃废债务和金融欺诈行为，逐步形成以道德为支持、产权为基础、法律为保障的社会信用制度。加大处置银行不良资产工作力度，保证不良贷款率下降３个百分点。积极推进农村信用社体制改革，促进农村信用社步入可持续发展的良性循环轨道。进一步改善金融服务，加强政府和社会多元出资的担保体系建设，增强信贷资金投放能力。

　　（九）加快完善就业服务和社会保障体系，切实维护人民群众的根本利益

　　以扩大就业和提高社会保障能力为重点，解决好关系人民群众利益的问题，努力提高人民生活水平。

　　坚持发展经济与扩大就业并举，千方百计促进就业和再就业。继续贯彻劳动者自主择业、市场调节就业和政府促进就业的方针，多渠道增加就业岗位，全年新增就业５万人。认真落实再就业优惠政策，建立小额贷款信用担保体系，降低申办个体工商户和中小企业的门槛，为下岗失业人员创业提供条件。继续搞好就业援助活动，重点安置“４０５０”等就业困难群体。加强劳动力市场和社区劳动保障工作平台建设，扩大就业服务网络覆盖范围。大力开展就业和创业培训及中介服务，建立企业与培训机构紧密联系、专业技能与市场需求接轨的培训机制。实施农村劳动力转移工程，通过开辟″三农″服务专栏等方式，加强对农民的再教育，突出加强对农村劳动力的培训和职业介绍，促进农村剩余劳动力转移。

　　以扩面提率为重点，进一步完善社会保障体系。继续巩固和完善社会保险扩面联动机制，严厉制裁各种拒保行为。扩大养老保险覆盖面，基本养老保险参保人数达到３５万人。加大保费征缴力度，养老保险费收缴率达到８５％。基本养老保险个人账户记清率、上解率、企业离退休人员养老金社会化发放率均保持１００％。加强失业人员管理，确保符合条件的失业人员享受失业保险待遇。逐步扩大城镇职工基本医疗保险，重点解决破产企业退休人员和困难企业职工参加医疗保险问题，增强医疗保障能力。健全和完善城乡居民最低生活保障制度，切实加强动态管理，努力实现应保尽保。积极发展商业保险，促进社会保障多元化。

　　深入开展扶贫帮困活动，认真解决好群众关注的热点、难点问题。完善扶贫帮困运行机制和管理体制，积极开展临时捐赠和社会救助活动，把社会救助纳入经常化和制度化的轨道。继续实施开发式扶贫，努力改善贫困地区生产生活条件。更加关注民生，深入了解群众疾苦，依法维护群众权益。认真解决动迁回迁、供水供暖和拖欠农民工工资等关系群众利益的突出问题，切实保障弱势群体的基本生活。

　　（十）协调发展科技、教育等项社会事业，实现社会全面进步

　　加大社会事业改革和投入力度，逐步建立以人的全面发展为中心的社会事业体系。

　　深入实施“科教兴市”战略，走“人才强市”之路。加快建设科技创新体系，积极搞好企校协作，促进科技成果转化为现实生产力。完善以市（县）、区为主的教育管理体制，统筹发展各级各类教育，强力推进“双高普九”规划的实施。积极发展高中教育和职业教育，鼓励社会力量办学。深入推进素质教育，加强师德师风建设。紧紧抓住培养、吸引、用好人才三个环节，加强人才资源开发，着重培养一批高素质科技、管理人才和技能型实用人才，形成尊重知识、尊重人才、尊重劳动、尊重创造的良好环境。加强人才市场建设，促进人才合理流动。

　　抓好文化、卫生、体育等项工作，协调发展社会事业。深化文化体制改革，促进公益性文化事业和经营性文化产业发展。加大“扫黄打非”力度，强化音像场所和网吧管理，繁荣和净化文化市场。积极推进广电事业发展，努力提高广播电视节目编播质量和收视率。加强公共卫生设施建设，优化卫生资源配置，提高医疗水平。稳步推进农村新型合作医疗和医疗救助制度试点工作，进一步完善突发公共卫生事件应急处理、疾病预防控制、医疗救治和社区卫生服务体系。加强食品卫生监督管理，确保群众饮食健康。强化人口和计划生育工作，稳定低生育水平，控制性别比例，提高人口素质，全年人口出生率和自然增长率分别控制在６．１５‰和１．７６‰以内。加强体育设施建设，广泛开展群众性体育活动，努力提高竞技体育水平。

　　加强精神文明建设，提高社会文明程度。积极倡导全民学习、终身学习，努力创建学习型社会。围绕打造“诚信和谐、务实创新”的城市精神，深入开展群众性精神文明创建活动，争创全国文明城市。加强社会公德、职业道德、家庭美德和未成年人思想道德教育，在全社会形成高尚的基本道德规范。

　　（十一）加强民主法制建设，推进依法治市进程

　　健全社会主义民主，完善社会主义法制，促进政治安定和社会稳定。

　　充分发扬民主，保障人民的民主权利。认真执行人大及其常委会的各项决议、决定，主动接受人大及其常委会的依法监督和政协的民主监督，办好人大代表建议和政协委员提案。坚持民主决策，实行听证制度，广纳民言，集中民意，体现民志，提高决策的科学化水平。推行政务公开、厂务公开和村务公开，增强工作透明度。加强同各民主党派、工商联、无党派人士的联系，认真贯彻党的民族宗教和侨务政策。重视并支持工会、共青团、妇联等群团组织的工作，完善村民自治和城市居民自治，做到充分尊重群众、充分依靠群众。

　　全面推进依法行政，努力建设法治政府。深入贯彻实施国务院《全面推进依法行政实施纲要》，制订依法行政五年规划。继续开展“四五”普法教育和依法治市宣传活动，在全社会形成学法、知法、守法的良好氛围。严格落实行政执法责任制、过错追究制和评议考核制，强化行政执法监督，维护公平和正义。

　　有效化解社会矛盾，保持社会稳定。认真落实信访工作责任制，畅通信访渠道，妥善解决各类矛盾。强化公安基础建设，充分发挥武装警察部队的职能作用，积极构建打防控一体化的社会治安长效机制，保持对各种犯罪的高压态势。严厉打击“法轮功”等邪教组织活动，做好国家安全工作。依法强化国防教育、国防动员、民兵预备役和双拥工作，进一步密切军政、军民关系。建立健全安全生产监管体系，遏制重特大事故的发生。

　　（十二）加快政府职能转变，建设为民、务实、清廉的服务型政府

　　加强政府自身建设，努力提高行政效能，营造宽松的发展环境。

　　提高政府执政能力，切实履行各项职责。坚持科学、民主、依法执政，不断深化对社会主义市场经济运行规律的认识，找准政府工作的突破口和切入点，解决好事关改革、发展、稳定的重大问题。提高统筹规划和综合协调水平，调动一切积极因素，确保政府各项工作有序进行。

　　始终牢记为人民服务的宗旨，切实做到勤政为民。坚持把群众利益放在首位，健全各级政府和领导干部联系群众制度，体察民情、珍惜民力、排解民忧，把改革的力度、发展的速度和社会可承受的程度统一起来，实现好、维护好、发展好人民群众的根本利益。把满足人的需求与发展作为政府工作的重点，多做雪中送炭之事，切实做到权为民所用、情为民所系、利为民所谋。

　　加快转变政府职能，进一步改善发展环境。着力解决政府职责和管理“错位”问题，切实履行经济调节、市场监管、社会管理和公共服务职能。稳步推进事业单位改革，逐步建立运转协调、服务高效的管理体制和运行机制。进一步下放市级管理权限，扩大市（县）、区经济社会管理自主权。加强软环境建设，努力营造高效务实的政务环境、公平竞争的市场环境、公正规范的法制环境和诚信和谐的社会环境。

　　不断改进工作作风，努力提高行政效能。改变会风和文风，减少迎来送往和事务性应酬。坚决克服形式主义和官僚主义，做到摸实情、说实话、办实事、求实效，力戒浮躁、浮浅、浮夸。加大督查、督办力度，搞好跟踪推进，及时协调解决工作中遇到的矛盾和问题，形成狠抓落实的良好风气。强化行政效能监察，不断提高办事效率和服务水平。

　　进一步加强廉政建设，树立公正廉洁的政府形象。发扬艰苦奋斗、勤俭节约的优良传统，认真落实廉洁自律的各项规定。建立有效的权力制约机制，进一步规范各级干部的行政行为，加大从源头上预防和治理腐败的力度。继续治理“三乱”行为，坚决纠正损害企业和群众利益的不正之风，努力建设人民满意政府。

　　各位代表，２００５年是充满希望的一年。让我们在市委的正确领导下，紧紧依靠全市人民的智慧和力量，开拓进取，真抓实干，为全面完成今年的各项任务，早日实现营口的全面振兴而努力奋斗！
